# Revisiting the balanced inventory of desirable responding: psychometric structure and personality correlates across heterogeneous groups

**DOI:** 10.3389/fpsyg.2026.1788770

**Published:** 2026-04-24

**Authors:** Anna M. Dåderman, Gerhard T. Eriksson, M. Susanna T. Fred, Anna Lundström, Sandra Pennbrant

**Affiliations:** 1 Department of Social and Behavioral Studies, University West, Trollhättan, Sweden; 2 Department of Health Sciences, University West, Trollhättan, Sweden

**Keywords:** big five, differential item functioning, honesty–humility, impression management, personality processes, self-deceptive enhancement, self-regulation, socially desirable responding

## Abstract

**Introduction:**

Socially desirable responding (SDR) encompasses both self-enhancing self-views and strategic self-presentation, yet debate persists regarding whether these tendencies reflect response distortion or substantive self-regulatory traits.

**Methods:**

The dataset included 130 individuals (19 with medical In a heterogeneous sample of inmates, nurses, managers, and working adults, we examined the structure and personality correlates of the 16-item short form of the Balanced Inventory of Desirable Responding (BIDR-6). Phase 1 evaluated dimensionality and item functioning using exploratory graph analysis, item response theory, differential item functioning, and confirmatory factor analysis.

**Results:**

Results supported the theorized two-factor structure comprising Self-Deceptive Enhancement (SDE) and Impression Management (IM), with largely comparable structural patterns and minimal item bias across groups. Phase 2 examined construct validity within Big Five and HEXACO frameworks. SDE was associated with lower Neuroticism and higher Extraversion and Conscientiousness, whereas IM was positively related to Agreeableness, Conscientiousness, and Honesty–Humility and negatively related to Neuroticism. These associations were largely consistent across correctional and occupational contexts, although modest group differences emerged, particularly for IM.

**Discussion/Conclusion:**

Overall, findings indicate that the BIDR-6 captures trait-linked self- and social-regulatory processes that generalize across diverse evaluative environments, supporting interpretations of SDR as more than mere response distortion.

## Introduction

Self-report questionnaires remain a cornerstone of personality and behavioral assessment but are vulnerable to socially desirable responding (SDR)—the tendency to present oneself favorably, consciously or unconsciously. Such tendencies can compromise construct validity, particularly in settings where reputational stakes are high. In correctional contexts, offenders may underreport negative traits to appear compliant or low risk (Hildebrand et al., 2018), whereas in occupational environments such as management or nursing, people may adjust responses to maintain a positive social image (Bäckström and Björklund, 2021). Reliable SDR measures are therefore essential for accurate interpretation of self-report data across diverse contexts.

Initially, SDR was identified as faking (e.g., Crowne and Marlowe, 1960) based on anecdotal and empirical evidence of the phenomenon (Edwards, 1953). Paulhus (1984) provided the foundational conceptualization of SDR as comprising two related but distinct dimensions—Self-Deceptive Enhancement (SDE) and Impression Management (IM)—and developed the BIDR to operationalize this distinction (1991; 2002). SDE represents an unconscious favorability bias closely related to narcissism (Paulhus and John, 1998). SDE items measure honest but inflated self-presentation, a positively biased but sincerely held self-view. IM items measure deliberate self-presentation to others. Although this two-component model is well supported, some studies have reported alternative structures or limited factorial robustness across populations (Gnambs and Kaspar, 2015). A systematic review by Perinelli and Gremigni (2016) further highlighted that many applied studies still treat SDR as a unitary bias rather than a construct with distinct substantive components, underscoring the need for clearer conceptual delineation.

In correctional samples, SDR’s role has been examined meta-analytically. Hildebrand et al. (2018) found small but reliable associations between SDE, IM, and self-reported dynamic risk factors, suggesting that SDR influences—but does not invalidate—offender self-reports. More broadly, meta-analytic evidence shows that SDR exerts modest, domain-specific influences on self-report validity (Gnambs and Kaspar, 2015; Kim and Kim, 2019).

Across studies, SDE correlates negatively with Neuroticism and positively with Extraversion and Conscientiousness, reflecting self-confidence and emotional resilience. IM, in contrast, is consistently associated with Agreeableness and Conscientiousness, and within the HEXACO framework also with Honesty–Humility (Bäckström and Björklund, 2021; Paulhus, 2002; Zettler et al., 2015). Importantly, these associations do not imply that IM reflects truthful or unbiased responding. Rather, as emphasized by Paulhus (1984, 2002), IM represents a strategic form of self-presentation aimed at maintaining social approval and avoiding negative evaluation. Traits such as Agreeableness, Conscientiousness, and Honesty–Humility may facilitate effective norm-conforming behavior, but in the context of IM they can also support selective self-disclosure, underreporting of undesirable behaviors, and overreporting of socially valued ones. Thus, IM appears best understood as an interpersonal self-regulation strategy that is socially adaptive yet potentially distorting in evaluative settings, rather than as a simple indicator of moral character.

Historically, socially desirable responding has often been conceptualized as a source of measurement error that contaminates self-report data. From this perspective, IM reflects deliberate distortion and SDE reflects unconscious positivity bias. However, subsequent theoretical developments have challenged the view of SDR as mere artifact. Empirical evidence has shown that SDE and IM display stable individual differences, systematic associations with personality traits, and predictive validity for behavioral outcomes. These findings have led to interpretations of SDR as reflecting substantive self-regulatory tendencies rather than solely response distortion (de Vries et al., 2013; Paulhus, 2002; Uziel, 2010).

The validity and utility of social desirability scales remain debated. Some reviews have questioned the substantive validity of social desirability scales, arguing that they may neither clearly capture response bias nor stable personality traits and, in some cases, recommending that scholars refrain from their use altogether (e.g., Lanz et al., 2022). Others caution that treating SDR purely as error may suppress substantively meaningful variance (Perinelli and Gremigni, 2016). In contrast, meta-analytic and empirical evidence indicates that SDE and IM show systematic trait associations and context-sensitive functioning, supporting their interpretation as psychologically meaningful constructs (de Vries et al., 2013; Hildebrand et al., 2018; Uziel, 2010). Within applied fields such as organizational and forensic psychology, several authors have argued that when SDR is theoretically relevant, instruments such as the BIDR should be examined rather than automatically controlled (Moorman and Podsakoff, 1992). Meta-analytic evidence suggests that controlling for impression management or self-deception does not substantially alter the predictive validity of personality measures (Li and Bagger, 2006), further complicating the view of SDR as mere response distortion. Thus, the role of the BIDR remains contested: it may function as a bias indicator, a personality-linked self-regulatory construct, or both, depending on context. Even if SDR reflects meaningful individual differences, questions remain regarding the BIDR’s structural robustness and generalizability across groups that differ substantially in social norms and evaluative pressure. Prior research has noted item-level anomalies and cross-loadings, particularly in heterogeneous samples (Gnambs and Kaspar, 2015; Perinelli and Gremigni, 2016).

Such inconsistencies raise questions about dimensional stability that cannot be resolved by factor-analytic approaches alone. Modern psychometric tools—such as network psychometrics and item response theory (IRT)—allow a more nuanced examination of dimensionality, item functioning, and cross-group comparability. Applying these approaches across heterogeneous contexts offers an opportunity to test whether SDR represents context-bound bias or structurally stable self-regulatory tendencies.

Several abbreviated versions of the BIDR have been proposed (Asgeirsdottir et al., 2016; Blasberg et al., 2014; Bobbio and Manganelli, 2011; Hart et al., 2015), reflecting different selection criteria, scoring and contexts. The present study uses the 16-item BIDR-6 Short Form by Bobbio and Manganelli (2011), which preserves the original SDE–IM distinction while reducing respondent burden. However, short-form versions of the BIDR have received comparatively limited comprehensive psychometric evaluation across heterogeneous applied groups.

While most foundational studies relied on student samples, later work has emphasized the need to examine SDR across applied contexts characterized by differing evaluative demands (de Vries et al., 2013; Uziel, 2010). Unlike prior studies that focused on student or single-occupational samples the present study builds on this tradition by evaluating the psychometric structure and nomological network of the BIDR-6 across correctional, healthcare, managerial, and general working populations using contemporary psychometric methods. We (a) examined the BIDR-6’s structure and measurement properties using exploratory graph analysis (EGA), IRT, and confirmatory factor analysis (CFA), and (b) assessed its construct validity through associations with Big Five and HEXACO personality traits. This integrative design enables a rigorous test of the BIDR’s psychometric stability and theoretical coherence across diverse social contexts.

### Theoretical framework

SDR has increasingly been conceptualized not merely as a measurement artifact but as a theoretically meaningful individual-difference construct reflecting self- and social-regulatory tendencies. Contemporary models conceptualize IM as a strategic, context-sensitive form of interpersonal self-regulation aimed at managing social impressions, whereas SDE reflects relatively stable intrapersonal biases that support affect regulation and self-esteem maintenance (Paulhus, 1984; Sedikides and Gregg, 2008; Uziel, 2010). These tendencies have been linked in prior research to outcomes such as cooperation, rule compliance, risk behavior, and interpersonal functioning, and they are connected to motivational and emotion-regulation systems. Thus, SDR may shape behavior in high-stakes environments—clinical, organizational, or correctional—rather than merely distorting questionnaire responses. Examining the dimensionality and correlates of SDR across distinct populations therefore offers insight into how people regulate their public and private selves and why these self-regulatory tendencies matter for well-being, relationships, and performance.

The conceptualization of SDE as an intrapersonal self-enhancement process and IM as interpersonal self-regulation is grounded in Paulhus (1984, 2002)’ original framework, as well as later extensions emphasizing emotion regulation and social control (Sedikides and Gregg, 2008; Uziel, 2010, 2014). Within this framework, SDE can be understood as an intrapersonal self-enhancement process that supports emotional stability and self-esteem maintenance, whereas IM reflects interpersonal self-regulation aimed at maintaining social approval and adherence to norms. Both processes may therefore have adaptive functions depending on context, even though they can also introduce distortion in self-report settings. Examining their structural stability and personality correlates across markedly different evaluative environments provides a way to test whether SDR reflects context-bound bias or more generalizable self-regulatory dispositions.

If SDE and IM are merely methodological artifacts, their internal structure and personality correlates should be unstable across contexts. In contrast, stable dimensionality and predictable trait associations would support interpreting them as meaningful psychological constructs. Accordingly, a rigorous structural and nomological evaluation of the BIDR-6 across diverse populations is theoretically informative beyond psychometric refinement.

### Aims and hypotheses

This study aimed to advance theoretical understanding of SDR by examining whether its two primary components—SDE and IM—reflect stable self-regulatory tendencies that generalize across diverse social and institutional contexts. To examine whether SDR operates consistently across contexts characterized by differing evaluative pressures, we included four socially and occupationally distinct groups: inmates (high external scrutiny and institutional control), nurses (normative and ethical regulation), managers (performance-oriented self-presentation), and working adults from the general population. This design allowed us to test whether SDE and IM function similarly across contexts varying in social pressure and institutional norms. Rather than treating SDR solely as measurement bias, we conceptualize SDE as an internal self-enhancement process and IM as an interpersonal self-regulation strategy shaped by situational demands. We therefore evaluated whether these theoretically grounded components (a) replicate across groups that vary substantially in evaluative pressure (e.g., inmates vs. occupational groups), and (b) show predictable relationships with core personality traits linked to self-control, emotional regulation, and social adaptation.

Based on these perspectives, we hypothesized that:

1 Two-component structure

The BIDR-6 would reproduce the theorized SDE–IM factor structure across all groups.

2 Generalizability of item functioning

Items would show adequate psychometric functioning and minimal differential item functioning (DIF), indicating that SDE and IM operate similarly across groups differing in social and institutional context.

3 SDE as internal self-enhancement

SDE would correlate negatively with Neuroticism, consistent with models describing self-deception as a stabilizing self-regulation mechanism.

4 IM as interpersonal self-regulation

IM would correlate positively with Agreeableness, Conscientiousness, and Honesty–Humility, consistent with theories linking IM to norm adherence and social adaptation.

### What this study contributes

Whereas much of the foundational work on the BIDR and its nomological network was conducted in student samples (Paulhus, 1991; Paulhus and John, 1998), this study contributes to the literature in three key ways. First, it provides a rigorous psychometric evaluation of the BIDR-6 using modern network and IRT methods across four demographically and contextually distinct populations. Second, it examines whether IM and SDE maintain their conceptual distinctiveness and interpretive utility across settings that differ markedly in social norms, behavioral expectations, and self-presentation pressures (e.g., prisons vs. workplaces). Third, it evaluates whether SDR shows meaningful associations beyond core personality traits, clarifying whether SDR reflects a substantive self-regulatory disposition rather than merely a methodological contaminant. Together, this approach offers a theoretically grounded examination of SDR as a self-regulatory system with implications for functioning across diverse real-world contexts.

Prior studies have examined the nomological network of the BIDR. The present contribution extends this work by (a) integrating modern psychometric approaches (EGA, IRT, DIF) with construct validation, (b) testing cross-context robustness across correctional and occupational settings, and (c) examining item-level functioning within these diverse environments.

## Methods

### Participants, settings, and procedures

The study included a total sample of *N* = 1,887 participants drawn from five groups representing distinct social contexts. These groups formed two analytically independent samples based on the personality inventory administered.

#### Big five sample (IPIP-NEO-120)

This sample consisted of:

- Inmates (*n* = 287) representing a correctional context
- Workers 1 (*n* = 162) serving as occupational controls to inmates

These two groups were used in analyses involving the IPIP-NEO-120 personality inventory.

#### HEXACO sample (Mini-IPIP6)

This sample consisted of:

- Managers (*n* = 344)
- Nurses (*n* = 939)
- Workers 2 (*n* = 171) serving as occupational controls

These three groups were used in analyses involving the Mini-IPIP6.

Managers, nurses, Workers 1, and Workers 2 represent diverse occupational contexts, whereas inmates represent a correctional context characterized by high external scrutiny and institutional regulation.

Data were collected between 2016 and 2019 primarily in southern and western Sweden using datasets originally gathered for prior research purposes.

#### Analytic strategy regarding pooling

Psychometric analyses evaluating the internal structure of the BIDR-6 (EGA, IRT, CFA) were conducted on the pooled dataset (*N* = 1,887) to maximize parameter stability and test cross-context robustness. DIF analyses were conducted to evaluate measurement equivalence across correctional and occupational contexts. Results indicated minimal item bias, supporting the appropriateness of pooled structural analyses.

In contrast, construct validation analyses (correlations and regressions) were conducted within the analytically relevant samples (Big Five vs. HEXACO), and sample type was modeled explicitly when theoretically relevant.

All groups exceeded *N* = 160, a recommended threshold for stable correlation estimates (Schönbrodt and Perugini, 2013).

Demographic characteristics are presented in Table 1. Detailed recruitment procedures are described in Supplementary material S1. Participation was voluntary and anonymous, and no immediate evaluative or institutional consequences were associated with responses.

**Table 1:** Demographic characteristics of study groups and data sources.

Group	*N*	Age (years) min–max, *M* (*SD*)	Women *n* (%)	Age Women (*SD*) / Men (*SD*)
Inmates	287	20–67, 36 (10.9)	74 (25.8)	36.5 (10.4)/35.6 (11.2)
Managers	344	23–65, 49 (8.6)	199 (58)	48.4 (9.0)/49.3 (8.0)
Nurses	939	23–70, 42 (11.9)	822 (87.5)	42.0 (11.9)/41.8 (11.8)
Working adults	333	19–78, 37 (11.3)	264 (79.3)	37.1 (11.1)/38.7 (12.2)
Workers 1	162	20–65, 35 (9.7)	141 (87)	34.1 (8.3)/34.8 (9.9)
Workers 2	171	19–78, 40 (12.1)	123 (71.9)	39.8 (11.8)/40.7 (13.1)

Total sample size was *N* = 1,887. Managers, nurses, Workers 1, and Workers 2 represent occupational contexts, whereas inmates represent a correctional context. Workers 1 (controls to inmates) and Workers 2 served as control groups within their respective analytic samples and are therefore reported separately.

Additional procedural details for all groups, including inmate recruitment, worker control sampling, and student thesis–based data collection, are provided in Supplementary material S1. All datasets were collected under the oversight of relevant university authorities and in accordance with applicable ethical standards.

Age data were incomplete for some participants: inmates (*n* = 11; 2.8%), managers (*n* = 3; 0.9%), and nurses (*n* = 212; 22.6%). Complete BIDR data were available for 271 inmates. For 16 inmates with missing SDE and IM scores, values were imputed using corresponding IPIP subscale scores.

### Power considerations

The present study relied on existing datasets collected for prior research purposes. Given the large overall sample size (*N* = 1,887) and substantial subgroup sizes (all exceeding *N* = 160), statistical power for detecting small-to-moderate effects in correlational and regression analyses was high. For example, a sample of *N* = 400 provides > 0.80 power to detect correlations as small as *r* = 0.14 at α = 0.05. The total sample and group sizes in the present study exceeded these thresholds. Moreover, the primary aim concerned psychometric evaluation (e.g., dimensionality, item functioning, and measurement stability), for which large samples are recommended to ensure stable parameter estimation. Accordingly, the available sample size was considered adequate for all planned analyses.

### Ethical considerations

The study was reviewed by the Regional Ethics Review Authority (DNR 2014/730 B22) and conducted in accordance with the Swedish Ethics Review Act (2003:460) and the Declaration of Helsinki. Under Swedish law, anonymous questionnaire-based studies that do not involve personal data relating to racial or ethnic origin, political opinion, religious or philosophical beliefs, trade union membership, health, sexual life, or sexual orientation are exempt from mandatory ethical board approval. This study fulfilled these criteria and therefore did not require formal approval.

Additional institutional permissions were obtained from the host universities, the Swedish Prison and Probation Service, and participating organizations. Data were collected using fully anonymous standardized questionnaires. Participants received written information about the study and provided informed consent by affirming a statement on the first page of the survey. No identifiable or sensitive personal data were collected, and participation was voluntary and could be discontinued at any time.

The study adhered to established ethical principles regarding informed consent, confidentiality, and responsible use of data. All procedures were carried out in accordance with institutional and national ethical standards and with the Declaration of Helsinki.

## Measures

### Social desirability: balanced inventory of desirable responding (BIDR-6) short form

The Swedish version of the Balanced Inventory of Desirable Responding (BIDR-6) Short Form used in this study was translated from the Italian BIDR-6 Short Form developed by Bobbio and Manganelli (2011), which itself was derived from the original BIDR (Paulhus, 1991). Items are rated on a 7-point scale (1 = *Strongly disagree*, 7 = *Strongly agree*). The BIDR-6 assesses two dimensions of socially desirable responding: SDE, reflecting positively biased but sincerely held self-views, and IM, reflecting deliberate self-presentation aimed at social approval (Kovačić, 2023; Paulhus, 2002). Item wording is presented in Table 2 in the Results section, alongside item discrimination parameters. Item scoring followed the version reported by Bobbio and Manganelli (2011). Item BIDR13 is not included in the Italian version; instead, it represents a Swedish adaptation of Item 22 (“I never cover up my mistakes”) from the original 40-item BIDR (Paulus, 1988, 1991), replacing an item referring to littering behavior that was not culturally appropriate in Sweden. The Swedish version does not apply Paulhus’s dichotomous scoring recommendation (“add one point for each response of 6 or 7”), but treats all items as continuous indicators.

**Table 2:** Item discrimination parameters for the Swedish version of the BIDR-6 Short Form estimated using the graded response model.

Dimension Item	Item text	*a*	Interpretation
SDE			
BIDR7	I am very confident in my judgements.	1.91	Very high
BIDR2	I always know why I like things.	1.45	High
BIDR6	I am a completely rational person.	1.42	High
BIDR4	I am fully in control of my own fate.	1.28	Moderate
BIDR5	I never regret my decisions.	1.19	Moderate
BIDR1	My first impression of people usually turn out to be right.	0.98	Moderate
BIDR3	Once I have made up my mind, other people can seldom change my opinion.	0.97	Moderate
BIDR8	It is all right with me if some people happen to dislike me.	0.67	Moderate
IM			
BIDR10	There have been occasions when I have taken advantage of someone. (R)	2.05	Very high
BIDR9	I sometimes tell lies, if I have to. (R)	1.96	Very high
BIDR15	I have taken sick-leave from work or school even though I was not really sick. (R)	1.28	Moderate
BIDR16	I have some pretty awful habits. (R)	1.13	Moderate
BIDR14	I have done things that I do not tell other people about. (R)	1.03	Moderate
BIDR12	I have said something bad about a friend behind his or her back. (R)	0.94	Moderate
BIDR11	I always obey laws, even if I am unlikely to get caught.	0.79	Moderate
BIDR13	I never try to hide my mistakes.	0.56	Low

*a* = item discrimination parameter. Items are ranked by their *a* values. Following conventional guidelines, values < 0.25 indicate very low discrimination, 0.25–0.64 low, 0.65–1.34 moderate, 1.35–1.68 high, and ≥ 1.69 very high. BIDR-6, Balanced Inventory of Desirable Responding; SDE, Self-Deceptive Enhancement; IM, Impression Management. (R), reversed score. Parameters were estimated using the graded response model in IRTPRO 4.2. Model fit: SDE—RMSEA = 0.03; IM—RMSEA = 0.03. We used a Swedish version (Dåderman and Kajonius, 2024) of the BIDR-6. Full item-level diagnostics, including thresholds, monotonicity indices, and standardized loadings, are provided in Supplementary Table S1.

Internal consistencies (Cronbach’s α) for the SDE and IM scales were as follows across groups: 0.79/0.73 (inmates), 0.70/0.67 (managers), 0.70/0.67 (nurses), 0.76/0.65 (Workers 1), and 0.74/0.62 (Workers 2). The correlation between SDE and IM in the total sample was negligible (*r* = 0.015, *p* = 0.506).

### Concurrent validity assessment

To evaluate the concurrent validity of the Swedish version of the BIDR-6, correlations were computed between its SDE and IM subscales and corresponding Swedish translations of the IPIP NEO PI-R SDE and IM scales (Bäckström, 2007) in two groups: inmates and Workers 1. Results indicated moderate to strong convergence between the BIDR-6 and IPIP measures. Specifically, SDE correlated *r* = 0.59 (*p* < 0.001) among inmates and *r* = 0.43 (*p* < 0.001) among Workers 1 with the IPIP SDE scale, while IM correlated *r* = 0.63 (*p* < 0.001) in both groups with the IPIP IM scale. The instruments are conceptually aligned but differ in format and operationalization; therefore, moderate-to-strong correlations are consistent with expectations for related but non-identical measures of socially desirable responding. These findings support the concurrent validity of the Swedish BIDR-6.

### Personality inventories

Two personality instruments were used in different data collections. The Big Five sample (inmates and Workers 1) completed the 120-item IPIP-NEO (Johnson, 2014). The HEXACO sample (managers, nurses, and Workers 2) completed the 24-item Mini-IPIP6 (Sibley et al., 2011), which includes Honesty–Humility in addition to the five broad domains. Both instruments assess broad personality traits using short descriptive statements. For conceptual clarity, Emotional Stability is treated as the inverse of Neuroticism; all hypotheses and results are therefore reported in terms of Neuroticism (higher scores = higher neuroticism).

#### IPIP-NEO-120

The IPIP-NEO-120 (Johnson, 2014) is a public-domain measure of the Five-Factor Model (Costa and McCrae, 2008) comprising 120 items—24 per domain (Agreeableness, Conscientiousness, Extraversion, Neuroticism, and Openness). Items are rated on a 5-point scale (1 = *Almost never*, 5 = *Almost always*). Internal consistencies (Cronbach’s α) ranged from 0.68–0.88 in inmates and 0.75–0.86 in Workers 1, consistent with prior validation work (Johnson, 2014).

#### Mini-IPIP6

The Mini-IPIP6 (Sibley et al., 2011), derived from Goldberg’s (1999) International Personality Item Pool and Donnellan et al. (2006), assesses six HEXACO traits: Agreeableness, Conscientiousness, Extraversion, Neuroticism, Openness, and Honesty–Humility. Items are rated on a 7-point scale (1 = *Strongly disagree*, 7 = *Strongly agree*). Consistent with prior findings (Woo et al., 2014), Openness showed relatively lower reliability but acceptable mean inter-item correlations (*M*_iic_). In the current study, Cronbach’s α/*M*_iic_ values ranged from 0.61–0.76/0.31–0.40 (managers), 0.59–0.73/0.23–0.40 (nurses), and 0.63–0.80/0.30–0.50 (Workers 2).

#### Data handling

Missing values (< 1%) were replaced with the respective scale mean, except where data loss exceeded 20% per participant. Three inmates were excluded from IPIP-NEO-120 analyses due to extensive missing data, though their other responses were retained. Descriptive statistics (*M*, *SD*) and internal consistency (α) were computed for all scales; for those with fewer than eight items, *M*_iic_ were also reported to support reliability evaluation.

Together, the IPIP-NEO-120 and Mini-IPIP6 provided comprehensive coverage of the Big Five and HEXACO personality domains, enabling analyses of the BIDR-6 dimensions (SDE and IM) in relation to established trait frameworks. The subsequent analytical strategy section outlines the procedures used to evaluate the psychometric properties of the BIDR-6 and to test its nomological validity across these personality measures.

## Analytical strategy

### Phase 1 – Psychometric evaluation (EGA, IRT, CFA)

Phase 1 analyses were conducted on the pooled dataset comprising all five groups and proceeded in three sequential steps to evaluate the internal structure and measurement properties of the BIDR-6.

First, the latent dimensionality of the BIDR was explored using EGA (Golino and Epskamp, 2017; Golino et al., 2020) based on the polychoric correlation matrix of the 16 items. EGA applies network psychometrics and community-detection algorithms to identify item clusters representing underlying latent dimensions. All analyses were performed in R using the EGAnet and psychTools packages.

Before estimating dimensionality, unique variable analysis (UVA) was used to identify locally dependent or redundant items (Christensen et al., 2023). After evaluating potential redundancies, EGA was conducted, and its dimensional stability was examined using bootEGA (Christensen and Golino, 2021).

To quantify item–dimension relationships, we computed revised network loadings (Christensen et al., 2025). Revised network loadings build on the original network loading algorithm by improving estimation accuracy and interpretational clarity, and they are directly comparable to factor loadings in traditional latent variable models. Together, these procedures allowed us to evaluate whether the BIDR items reproduced the theorized two-factor structure—SDE and IM.

Second, IRT analyses were conducted using the graded response model (GRM; Samejima, 1969) in IRTPRO (Version 4.2; Cai et al., 2011). The BIDR consists of two distinct dimensions. Each dimension was analyzed separately using unidimensional IRT to estimate item parameters and latent trait scores. This approach allows precise modeling of each dimension while maintaining interpretability for established scoring practices. Analyses were conducted following standard procedures in psychometric research (de Ayala, 2009; Embretson and Reise, 2000). Item fit and model assumptions were evaluated using recommended diagnostics for unidimensional IRT models. Prior to model estimation, we evaluated the GRM assumptions of monotonicity, local item independence, and approximate unidimensionality.

- *Monotonicity* was assessed with the Mokken R package to verify that the probability of endorsing higher response categories increased with higher latent trait levels (θ).
- *Local item independence* was examined via IRTPRO’s standardized LDχ^2^ statistics. Although some item pairs showed moderate residual dependence, none exceeded critical thresholds, and no items were removed. Item wording was also reviewed to ensure conceptual distinctness.
- *Approximate unidimensionality* was examined using the two-factor structure identified by EGA, and separate GRM models were estimated for the SDE and IM dimensions under the assumption of local unidimensionality.

To evaluate cross-group measurement equivalence, DIF analyses were performed with inmates as the focal group and managers, nurses, and working adults as the reference group. Likelihood-ratio χ^2^ tests compared models constrained and unconstrained on discrimination (χ^2^_a_) and difficulty (χ^2^c/a) parameters.

Finally, CFAs were conducted to compare alternative structural models of the BIDR-6, particularly regarding the placement of Item BIDR8. Analyses employed robust maximum likelihood estimation (MLR) using the lavaan package in R. Model adequacy was evaluated with the Comparative Fit Index (CFI), Tucker–Lewis Index (TLI), Root Mean Square Error of Approximation (RMSEA), and Standardized Root Mean Square Residual (SRMR), applying conventional cutoffs (CFI and TLI ≥ 0.90; RMSEA and SRMR ≤ 0.08; Hu and Bentler, 1999).

It is worth noting that CFAs on complex instruments rarely achieve ideal model fit and often require multiple model tests, compromises, and extensive modifications, especially when comparing factor structures across cultures and samples (Byrne and Watkins, 2003; Furr, 2022).

This multi-step approach provided a comprehensive evaluation of the BIDR’s internal structure, item-level performance, and cross-group comparability, forming the empirical foundation for the construct validation analyses in Phase 2.

### Phase 2: Construct validation – regression and group effects

Phase 2 examined the nomological validity of the BIDR-6 by relating SDE and IM to major personality dimensions. Because participants completed different personality inventories, analyses were conducted separately for the Big Five sample and the HEXACO sample.

Bivariate Pearson correlations were computed between BIDR scales and personality traits for the IPIP-NEO-120 (Big Five) sample and for the Mini-IPIP6 (HEXACO) sample. The Mini-IPIP6 additionally includes Honesty–Humility, which was analyzed alongside the Big Five traits to capture the broader HEXACO framework.

To examine the unique contributions of personality traits to SDE and IM, linear regression analyses were conducted separately within the Big Five and HEXACO samples using SPSS Version 31. Personality traits, age, and sex were entered simultaneously as predictors. Standardized beta coefficients (β) are reported.

To evaluate whether group membership contributed additional variance beyond personality traits, General Linear Models (GLM, univariate) were estimated. In these models, personality traits and age were treated as covariates, whereas group (inmates vs. Workers 1 in the Big Five sample; managers, nurses, and Workers 2 in the HEXACO sample) and sex were specified as fixed factors. This approach allowed simultaneous estimation of trait effects (continuous predictors) and categorical group differences without requiring manual dummy coding. Where omnibus group effects were significant in GLM analyses, follow-up one-way ANOVAs with Scheffé-adjusted *post hoc* comparisons were conducted to identify specific group differences.

This combined modeling strategy allowed us to (a) estimate trait-level nomological associations via regression coefficients, and (b) evaluate contextual group differences via adjusted mean comparisons, while maintaining statistical coherence across samples.

## Results

The analyses proceeded in two main phases. Phase 1 evaluated the internal structure and psychometric properties of the BIDR-6, including dimensionality, item functioning, and factorial validity. Phase 2 examined construct validity by testing associations between the BIDR dimensions and personality traits within the Big Five and HEXACO samples.

Psychometric analyses (EGA, IRT, CFA) were conducted on the pooled dataset comprising all five groups to maximize parameter stability and evaluate cross-group functioning through DIF. In contrast, construct validation analyses (correlations and regressions) were conducted separately within the Big Five and HEXACO samples.

For DIF testing, all occupational groups (managers, nurses, Workers 1, and Workers 2) were combined to form a pooled reference group, allowing stable two-group comparisons given restricted response variation in the manager group. Accordingly, DIF analyses compared inmates with the pooled occupational group.

Together, these analytic strategies allowed evaluation of structural robustness, measurement comparability, and nomological validity across distinct social contexts.

### Phase 1: Psychometric analyses

Phase 1 evaluated the dimensional structure and item functioning of the BIDR-6 using EGA, IRT, and CFA. Analyses were conducted on the pooled dataset to assess structural robustness across groups. Results are presented in the sequence EGA, IRT, and CFA, reflecting the progression from exploratory to confirmatory evaluation.

#### Exploratory graph analysis

**Figure 1:**
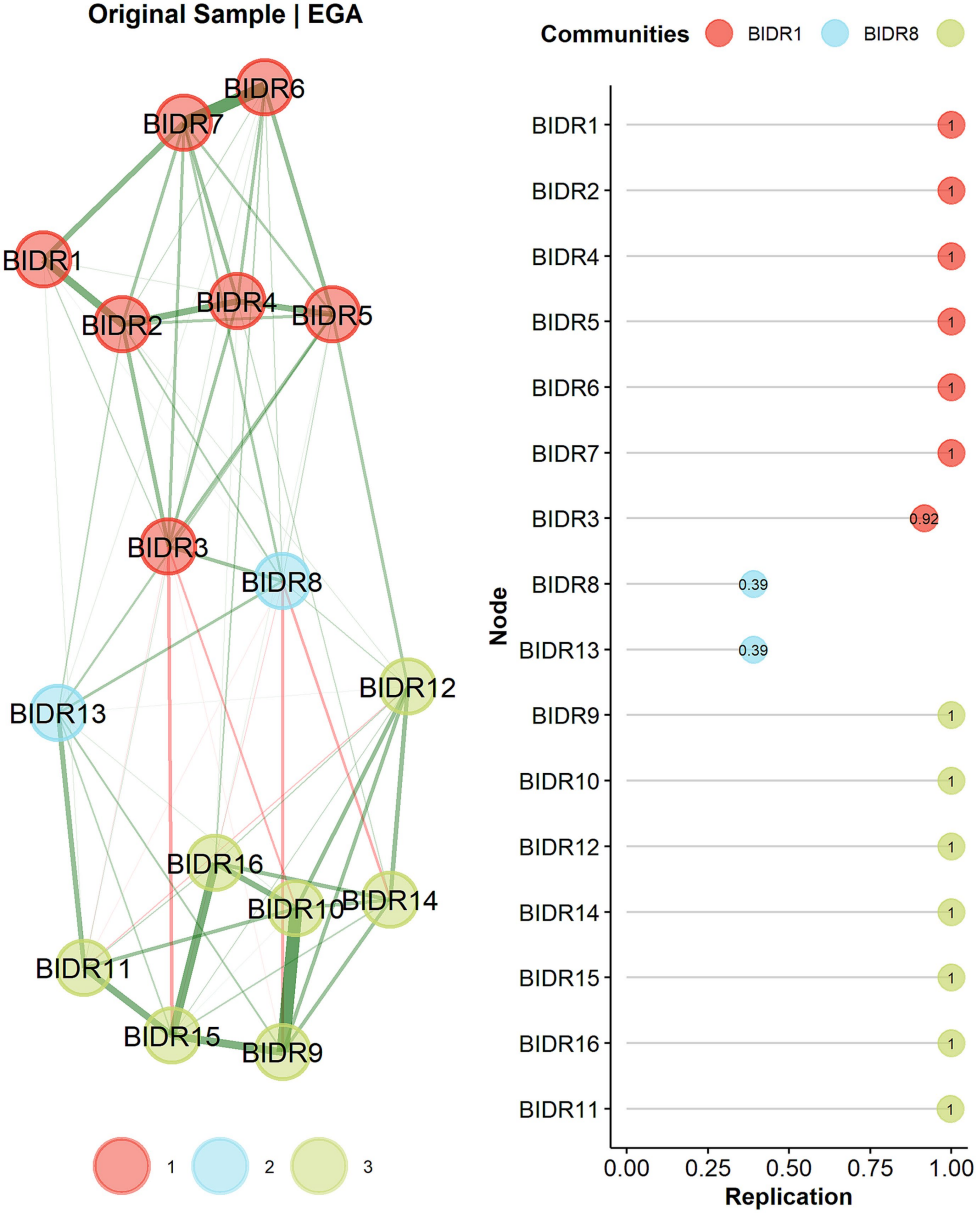
Exploratory graph analysis (EGA) network structure and bootstrap replication stability for the BIDR-6. The network graph displays the community structure identified by EGA, with nodes representing BIDR-6 items and edges representing partial correlations between items. Two distinct communities emerged, corresponding to Self-Deceptive Enhancement (SDE) and Impression Management (IM). Bootstrap EGA results indicate high structural stability for most items across replications, supporting the robustness of the two-dimensional solution. Items with lower replication stability reflect weaker or more ambiguous community assignment.

The EGA solution identified two dimensions consistent with prior research on the BIDR (Paulhus, 1991). However, item BIDR8 (“It is all right with me if some people happen to dislike me”) tended to align with the IM community rather than SDE and showed ambiguous network placement and weaker stability (Figure 1).

Figure 1 illustrates the EGA network structure and Bootstrap EGA replication stability. Two well-defined communities emerged, corresponding to SDE and IM. Bootstrap replication was consistent with the robustness of this configuration, with most items demonstrating high stability (> 0.90), except BIDR8 and BIDR13, which showed weaker stability and less consistent community assignments.

As shown in Table 3, all items—except BIDR8 and BIDR13—displayed moderate to strong primary loadings on their respective communities, supporting the expected two-factor solution. BIDR8 demonstrated weak and ambiguous associations with both dimensions, and BIDR13 showed a similarly low loading, suggesting limited contribution to construct representation.

**Table 3:** Revised network loadings for the BIDR-6 derived from exploratory graph analysis.

Item	Community 1 (SDE)	Community 2 (IM)	Psychometric problem
BIDR7	0.60	−0.05	Local dependence
BIDR2	0.50	0.01	
BIDR4	0.43	0.02	
BIDR6	0.40	0.04	Local dependence
BIDR5	0.36	0.07	
BIDR3	0.29	−0.17	
BIDR1	0.28	0.01	
BIDR9	−0.01	0.63	Local dependence
BIDR10	−0.03	0.58	Local dependence
BIDR15	−0.05	0.50	
BIDR16	0.04	0.40	
BIDR14	0.02	0.38	
BIDR11	0.01	0.31	
BIDR12	0.06	0.25	
BIDR13	0.06	0.12	Low loading
BIDR8	0.17	−0.05	Low loading

Loadings represent revised network loadings (Christensen et al., 2025), which are refined estimates derived from the original network loading algorithm and are interpretable in a manner similar to factor loadings. Following Christensen et al. (2025), values of 0.20 (small), 0.35 (moderate), and 0.50 (large) correspond approximately to traditional factor loadings of 0.40, 0.55, and 0.70, respectively. Local dependence was evaluated using Unique Variable Analysis (UVA; Christensen et al., 2023), with a cutoff of 0.25 shared variance indicating potential redundancy. Community structure and loadings were estimated using Exploratory Graph Analysis (EGA), with stability assessed via bootEGA (Christensen and Golino, 2021). Items flagged for local dependence or low loadings were retained based on conceptual distinctiveness and theoretical relevance.

UVA indicated local dependence for four items (BIDR7, BIDR6, BIDR9, BIDR10), with redundancy recommendations to retain BIDR6 and BIDR10. Given conceptual distinctiveness and lack of content overlap, all items were retained for further analyses.

Following identification of this network-derived structure, graded response IRT models were estimated separately for the SDE and IM dimensions to evaluate item-level functioning, including discrimination, thresholds, monotonicity, and local independence.

#### Item response theory

To evaluate the ambiguity observed for BIDR8, we conducted IRT analyses (de Ayala, 2009; Embretson and Reise, 2000). Model assumptions were checked prior to estimation. Item response curves showed monotonic relationships with θ, LDχ^2^ statistics indicated acceptable local independence, and the EGA-supported two-factor solution justified estimating separate unidimensional GRM models for SDE and IM.

When BIDR8 was assigned to the EGA-suggested IM dimension, it produced a negative discrimination parameter (*a* = −0.34), indicating that responses were not aligned with the underlying IM trait. When retained within the theoretically expected SDE dimension, its discrimination was positive and acceptable (*a* = 0.67).

Accordingly, Table 2 reports the GRM results estimated separately for SDE (with BIDR8 retained) and IM.

The GRM was estimated independently for the two dimensions identified in the EGA, and fit indices indicated that both models adequately represented the data. Full graded-response item parameters and diagnostics are presented in Supplementary Table S1.

Discrimination parameters ranged from low to very high. BIDR8 (SDE) and BIDR13 (IM) showed comparatively lower discrimination, but remained within acceptable limits and did not materially affect scale functioning. Overall, IM items tended to show slightly higher discrimination than SDE items.

**Figure 2:**
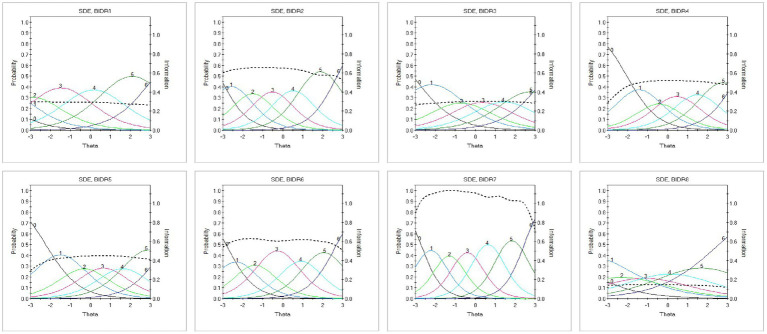
Item trace lines for the self-deceptive enhancement (SDE) dimension estimated using the Graded Response Model (GRM). Each panel shows category response curves for one SDE item, illustrating the probability of endorsing each response category across levels of the latent trait (θ). Most items display steep, well-ordered curves, indicating adequate discrimination and monotonic increases in response probability across the trait continuum. The figure illustrates how SDE items function across low to high levels of self-deceptive enhancement.

**Figure 3:**
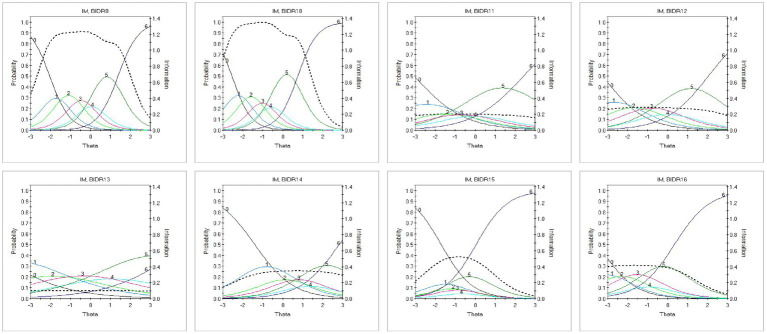
Item trace lines for the impression management (IM) dimension estimated using the Graded Response Model (GRM). Each panel presents category response curves for one IM item, showing how response probabilities vary as a function of the latent impression management trait (θ). Compared to SDE, several IM items show flatter slopes, reflecting lower discrimination. Response thresholds are generally ordered, indicating appropriate category functioning across levels of impression management.

Threshold parameters (*b*₁–*b*₆) were broadly ordered and covered a wide range of the latent continuum, suggesting good item spread. Monotonicity and local independence assumptions were generally supported, although a small number of item pairs showed mild residual dependence. Within the SDE dimension, BIDR8 showed the lowest discrimination, consistent with its relatively weaker network loading in the EGA. Similarly, BIDR13 showed the lowest discrimination within IM. Despite these comparatively lower parameters, both items were retained due to their conceptual relevance and nonredundant wording. These results are illustrated in Figures 2, 3.

Figure 2 shows the item trace lines for the SDE dimension. Most items exhibited steep, non-crossing curves, indicating strong discrimination and well-ordered thresholds. This pattern supports the monotonicity assumption and suggests effective functioning of the SDE items across the latent continuum.

Figure 3 displays the trace lines for the IM dimension. Although overall model fit was acceptable, several items showed somewhat flatter slopes, consistent with the wider variability in discrimination values reported in Table 2. The curves were generally ordered, indicating appropriate threshold spacing across response categories (Supplementary Table S1).

**Figure 4:**
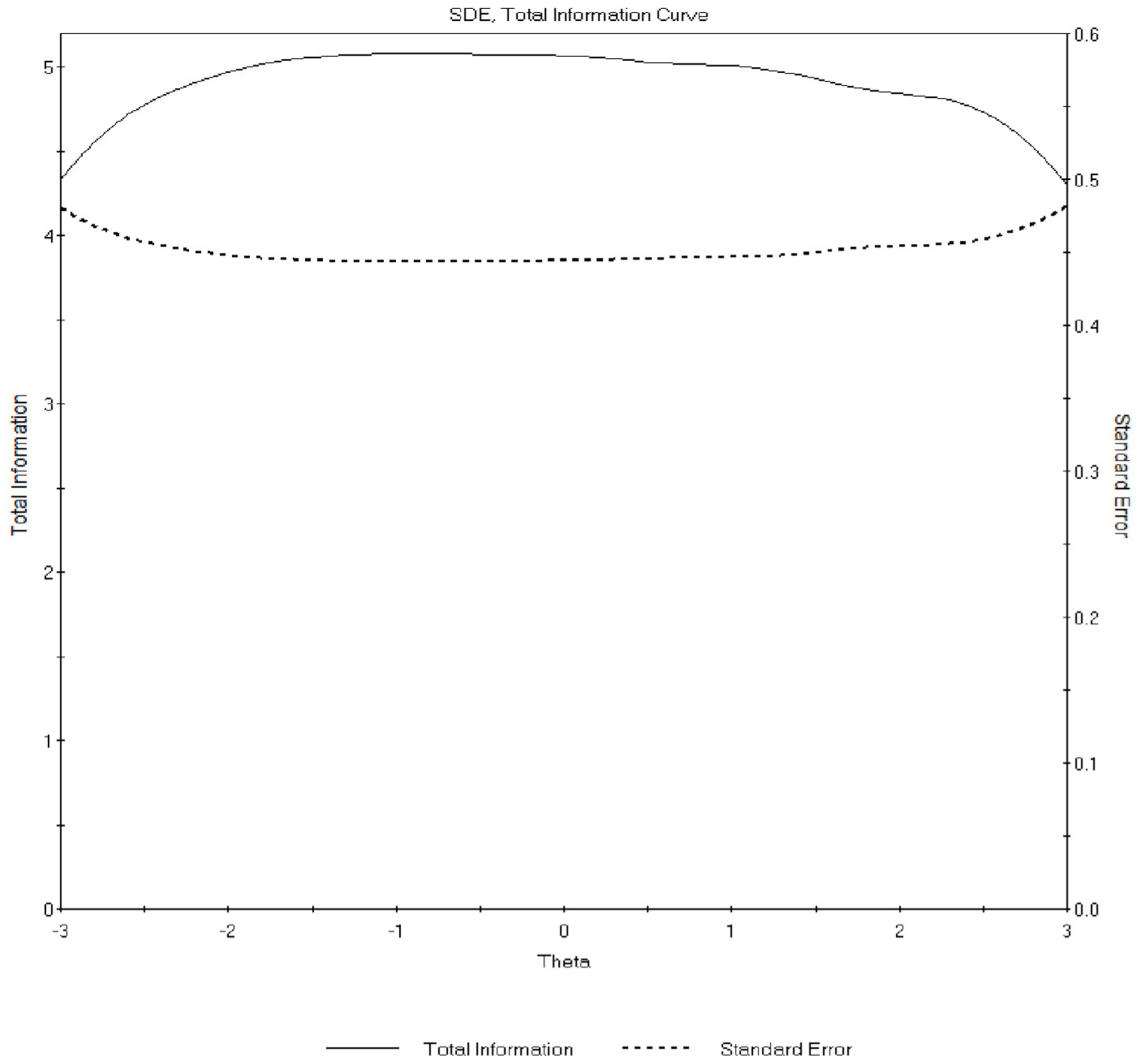
Total information curve (TIC) for the self-deceptive enhancement (SDE) dimension estimated under the GRM. The curve depicts measurement precision across the latent SDE continuum. Information is relatively high and broadly distributed from low to high trait levels, indicating stable precision across a wide range of self-deceptive enhancement. Peak information occurs around slightly below-average trait levels, corresponding to high conditional reliability.

**Figure 5:**
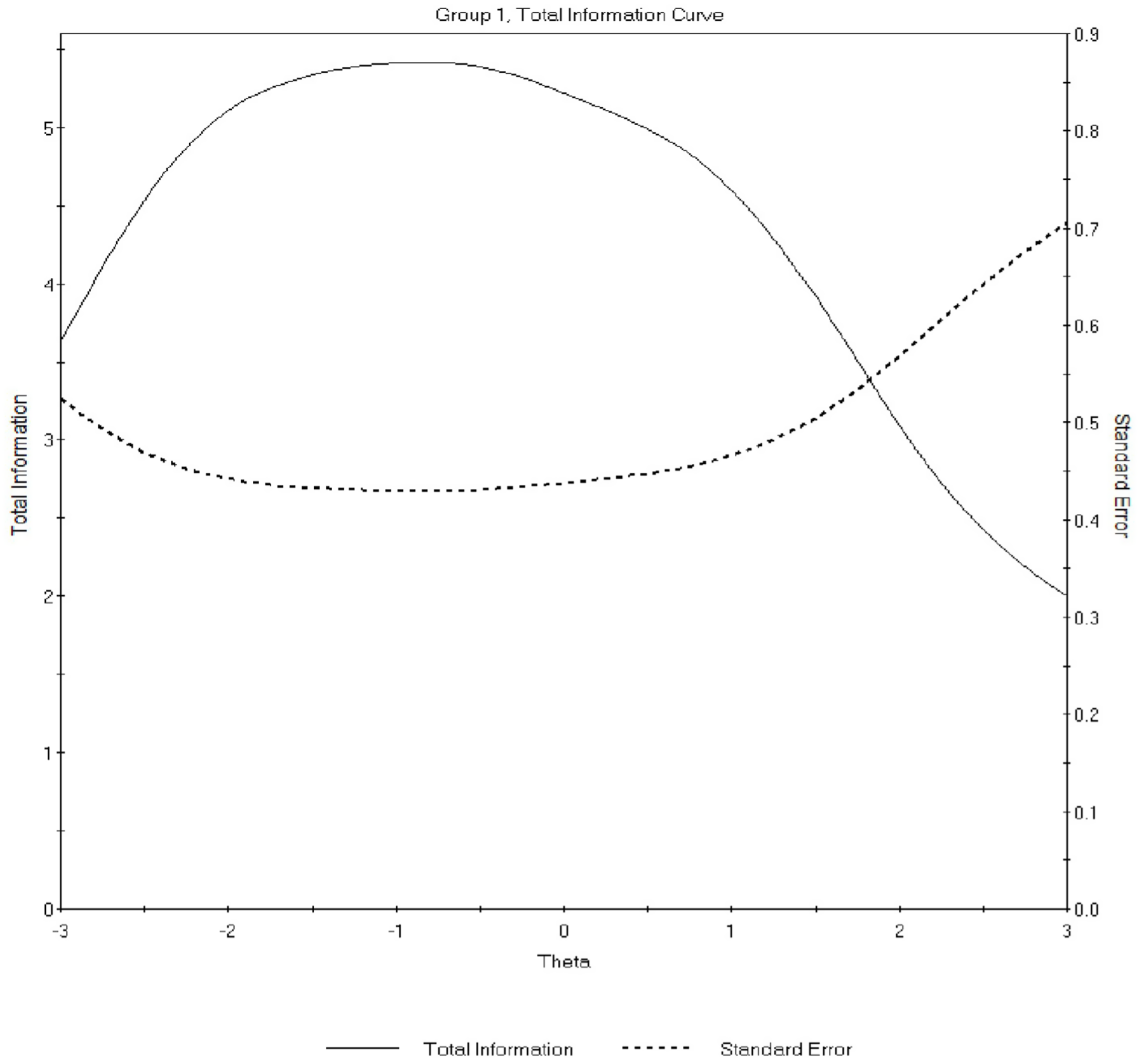
Total information curve (TIC) for the impression management (IM) dimension estimated under the GRM. The curve illustrates measurement precision for the IM dimension across the latent trait continuum. Information peaks at low-to-moderate levels of impression management and declines at higher trait levels, indicating that the IM scale is most precise for respondents with lower to average tendencies toward impression management.

Figures 4, 5 present the total information curves (TICs) for the SDE and IM dimensions, respectively. The SDE TIC was broad and relatively flat across the latent range (θ ≈ −3 to +3), with peak information around θ = −0.8 (max ≈ 5.09; conditional reliability ≈ 0.84). The IM TIC peaked near θ = −1.0 (max ≈ 5.41; conditional reliability ≈ 0.84) and declined more steeply at higher levels of the trait, indicating greater precision at low-to-moderate levels of IM. Marginal reliabilities were 0.80 (SDE) and 0.78 (IM).

Overall, the IRT findings supported satisfactory item discrimination and information, with SDE showing more uniform precision across trait levels and IM providing stronger precision in the lower-to-average range. Patterns were similar across subgroups.

#### Differential item functioning

To further examine measurement equivalence across groups, DIF analyses were conducted using inmates as the focal group and managers, nurses, and working adults as the reference group. Item BIDR8 did not display significant DIF in the discrimination parameter (*a*), indicating that its ability to differentiate people along the underlying trait was comparable across contexts. Although several items showed differences in difficulty parameters (*b*), only BIDR2 exhibited statistically significant DIF in discrimination (χ^2^_a_). Overall, this pattern suggests that BIDR8 operates similarly across occupational and correctional populations.

To further evaluate structural validity and to formally test the dimensional configuration suggested by the EGA and IRT results, CFAs were estimated next, with particular attention to the placement of BIDR8.

#### Confirmatory factor analyses

Two models were compared using CFA: (1) a structure in which BIDR8 was assigned to the EGA-suggested IM dimension, and (2) a structure in which BIDR8 was retained within the theoretically expected SDE dimension. Model 2 showed somewhat better fit than Model 1. Specifically, Model 2 yielded slightly higher fit indices (CFI = 0.83, TLI = 0.80) and lower error indices (RMSEA = 0.07, SRMR = 0.07) than Model 1 (CFI = 0.81, TLI = 0.77, RMSEA = 0.08, SRMR = 0.08).

Although this represents an incremental improvement, overall fit remained below conventional thresholds for good model fit (Hu and Bentler, 1999). Thus, CFA results do not provide decisive evidence but are more consistent with retaining BIDR8 within the SDE dimension rather than reassigning it to IM.

Taken together with the EGA, DIF, and IRT findings, these results suggest that BIDR8 is better understood as reflecting a form of self-deceptive self-assurance rather than impression management, consistent with the original conceptualization by Paulhus (1991) and the short-form adaptation by Bobbio and Manganelli (2011).

#### Summary of psychometric evidence

Taken together, the combined evidence from EGA, IRT, CFA, and DIF analyses is broadly consistent with the structural validity and cross-group robustness of the BIDR in this heterogeneous set of groups (inmates, managers, nurses, and working adults). EGA supported the expected two-dimensional structure of SDE and IM, and subsequent analyses clarified that BIDR8 is most coherently represented within the SDE dimension. IRT results indicated acceptable discrimination for BIDR8 only when specified as part of SDE, and CFA showed comparatively better—though still imperfect—fit under this specification.

Moreover, the absence of significant DIF in discrimination for BIDR8 across groups suggests that the item operates similarly in correctional and occupational contexts. Collectively, these findings indicate that the BIDR’s dimensional structure appears stable across diverse groups and that BIDR8, despite its initially ambiguous network placement, is most plausibly interpreted as reflecting SDE rather than IM.

### Phase 2: Construct validation

Phase 2 tested the theoretical coherence of the BIDR-6 dimensions by relating SDE and IM to major personality traits across the Big Five and HEXACO frameworks, building on the dimensional structure established in Phase 1. To balance interpretability with parsimony, correlations are presented using theoretically motivated groupings. For the IPIP-NEO-120, results are shown separately for inmates and for the combined inmate and Workers 1 group, allowing direct comparison of a high-stakes correctional context with a non-incarcerated reference group. For the Mini-IPIP6, correlations are shown for nurses and for the pooled occupational group (managers, nurses, and Workers 2), reflecting shared professional evaluation contexts. This approach avoids unnecessary fragmentation of the data while preserving the ability to detect meaningful group-specific patterns.

#### Correlations with big five and HEXACO traits

Consistent with prior research, SDE was expected to relate positively to Extraversion and negatively to Neuroticism, whereas IM was expected to relate positively to Agreeableness, Conscientiousness, and—within the HEXACO framework—Honesty–Humility. Correlations were examined separately within the Big Five and HEXACO samples (Tables 4, 5).

**Table 4:** Pearson correlations between BIDR dimensions, age, sex, and IPIP-NEO-120 traits in the big five sample (inmates and Workers 1).

Variables	SDE	IM	N	E	O	A	C	Sex	Age
**SDE**		**0.03**	−0.39*	0.37*	**−0.07**	−0.34*	0.40*	−0.19	−0.02
**IM**	**−0.02**		−0.23*	**−0.11**	**0.04**	0.42*	0.34*	−0.02	0.09
N	−0.32*	−0.23*		−0.31*	0.02	0.01	−0.61*	0.21*	−0.03
E	0.36*	**−0.13**	−0.33*		0.16	−0.24*	0.28*	−0.02	−0.22*
O	**−0.07**	**0.07**	0.002	0.11		0.31*	0.11	0.15	−0.03
A	−0.32*	0.49*	−0.02	−0.28*	0.32*		0.11	0.10	0.06
C	0.30*	0.38*	−0.51*	0.15*	0.11	0.26*		0.01	0.04
Sex	−0.18*	0.19*	0.19*	−0.19*	0.19*	0.37*	0.17*		0.03
Age	0.02	0.12	−0.09	−0.07	0.04	0.06	0.05	0.01	

N, neuroticism; E, extraversion; O, openness; A, agreeableness; C, conscientiousness.

*p < 0.05 after Bonferroni correction (0.05/38 = 0.00131). Sex: 1 = male, 2 = female. Correlation coefficients below the diagonal represent relationships in the combined inmate and Workers 1 group (*n* = 449). Correlation coefficients above the diagonal are specific to inmates (*n* = 287). Group sizes vary across analyses due to missing data for sex and age. Bold values indicate correlations between the BIDR dimensions (SDE and IM) and the focal personality variables.

The correlation between SDE and Neuroticism was significantly stronger in inmates (*r* = −0.39) than in Workers 1 (*r* = −0.16), *z* = −2.53, *p* = 0.006. The correlation between SDE and Conscientiousness was also stronger in inmates (*r* = 0.40) than in Workers 1(*r* = 0.23), *z* = 1.87, *p* = 0.031. The correlation between SDE and sex was stronger in inmates (*r* = −0.19) than in Workers 1(*r* = 0.07), *z* = −2.59, *p* = 0.005. Conversely, the correlation between IM and sex was stronger in Workers 1 (*r* = 0.23) than in inmates (*r* = −0.02), *z* = −2.49, *p* = 0.006.

**Table 5:** Pearson correlations between BIDR dimensions, age, sex, and Mini-IPIP6 traits in the HEXACO sample (managers, nurses, and Workers 2).

Variables	SDE	IM	N	E	O	A	C	H-H	Sex	Age
**SDE**		**0.10**	−0.20*	0.11*	**0.03**	**−0.03**	0.17*	−0.12*	−0.06	−0.03
**IM**	0.12*		−0.17*	−0.11*	**−0.07**	**0.02**	0.23*	0.22*	0.07	0.16*
N	−0.25*	−0.21*		−0.11*	−0.02	−0.04	−0.19*	−0.26*	0.10	−0.17*
E	0.14*	**−0.07**	−0.09*		0.29*	0.23*	0.06	−0.09	0.01	−0.07
O	**0.04**	**−0.06**	−0.02	0.26*		0.28*	0.02	0.02	−0.04	−0.10
A	**−0.02**	**0.07**	−0.03	0.25*	0.27*		0.13*	0.07	0.18*	−0.09
C	0.19*	0.27*	−0.18*	0.06	−0.02	0.11*		0.13*	0.14*	0.01
H-H	−0.11*	0.27*	−0.26*	−0.09*	0.02	0.11*	0.11*		0.07	0.15*
Sex	−0.13*	0.07	0.16*	0.02	0.01	0.21*	0.11*	0.05		0.01
Age	0.06	0.18*	−0.24*	−0.04	−0.06	−0.07	0.04	0.18*	−0.09*	

N, neuroticism; E, extraversion; O, openness; A, agreeableness; C, conscientiousness; H–H, honesty–humility.

*p < 0.05 after Bonferroni correction (0.05/38 = 0.00131). Sex: 1 = male, 2 = female. Correlation coefficients below the diagonal represent relationships in the combined managers, nurses, and Workers 2 sample (*n* = 1,454). Correlation coefficients above the diagonal are specific to nurses (*n* = 939). Group sizes vary across analyses due to missing data for sex and age. Bold values indicate correlations between the BIDR dimensions (SDE and IM) and the focal personality variables.

The correlation between SDE and IM was significantly stronger in nurses (*r* = −0.10) than in controls (*r* = −0.05), *z* = −1.86, *p* = 0.032. The correlation between SDE and Extraversion was significantly stronger in controls (*r* = −0.26) than in nurses (*r* = −0.11), *z* = −1.92, *p* = 0.027.

#### Big five sample

Table 4 shows correlations between BIDR subscales (SDE and IM) and IPIP-NEO-120 personality traits across correctional and occupational contexts. Below the diagonal are combined inmate and Workers 1 correlations; above are inmate-specific correlations. Most correlations were similar across groups, with two exceptions noted in Table 4.

#### HEXACO sample

Table 5 shows correlations between BIDR subscales (SDE and IM) and Mini-IPIP6 traits across occupational contexts for managers, nurses, and Workers 2. Below the diagonal are combined group correlations; above are nurse-specific correlations. Most correlations were similar between nurses and the combined data set (managers, nurses, and Workers 2), with two exceptions noted in Table 5.

Overall, correlation patterns were broadly consistent with predictions and highly similar across samples. Where differences emerged, they were limited to effect size rather than direction. In both Big Five and HEXACO samples, SDE correlated negatively with Neuroticism (IPIP-120: *r* = −0.33, *p* < 0.001; Mini-IPIP6: *r* = −0.29, *p* < 0.001), indicating that higher SDE is associated with lower reported neurotic symptoms. IM correlated positively with Agreeableness and Conscientiousness across instruments (e.g., IPIP-120: Agreeableness *r* = 0.22, *p* < 0.01; Conscientiousness *r* = 0.18, *p* < 0.01).

### Regression analyses and group effects

#### Big five sample

##### Self-deceptive enhancement

A multiple linear regression examined whether the Big Five traits, sex, and age predicted SDE scores. The predictors jointly explained 29.5% of the variance in SDE, *R*^2^ = 0.295, *F*(7,427) = 25.47, *p* < 0.001. Higher Extraversion (β = 0.18, *p* < 0.001) and Conscientiousness (β = 0.32, *p* < 0.001) were associated with higher SDE scores, whereas Agreeableness was negatively related to SDE (β = −0.33, *p* < 0.001). Neuroticism showed a negative but non-significant association (β = −0.09, *p* = 0.10). Openness, sex, and age were not significant predictors.

To examine adjusted group differences, a GLM was conducted with group (inmates vs. Workers 1) and sex as fixed factors and personality traits and age as covariates. Group membership did not significantly predict SDE after controlling for traits and demographics, *F*(1, 425) = 0.16, *p* = 0.688, indicating that trait–SDE associations were consistent across correctional and occupational contexts.

##### Impression management

A parallel regression showed that the predictors explained 33.0% of the variance in IM, *R*^2^ = 0.33, *F*(7,427) = 30.07, *p* < 0.001. Agreeableness (β = 0.42, *p* < 0.001) and Conscientiousness (β = 0.21, *p* < 0.001) were significant positive predictors, whereas Neuroticism was negatively associated with IM (β = −0.13, *p* = 0.011). Extraversion, Openness, sex, and age, were nonsignificant.

The GLM analysis indicated no significant group effect after covariate adjustment, *F*(1, 425) = 0.99, *p* = 0.321, again suggesting stable trait–IM associations across contexts.

#### HEXACO sample

##### Self-deceptive enhancement

A regression analysis examined HEXACO traits, sex, and age as predictors of SDE. The model explained 14.2% of the variance, *R*^2^ = 0.142, *F*(8,1,229) = 25.51, *p* < 0.001. Higher Conscientiousness (β = 0.18, *p* < 0.001) and Extraversion (β = 0.08, *p* = 0.004) were predicted higher SDE, whereas Neuroticism (β = −0.24, *p* < 0.001), Honesty–Humility (β = −0.16, *p* < 0.001), and sex (β = −0.09, *p* = 0.001) were negatively associated with SDE. Openness, Agreeableness, and age were not significant predictors.

A GLM analysis revealed a significant group effect, *F*(2, 1,225) = 32.98, *p* < 0.001, ηp^2^ = 0.051, indicating moderate differences across occupational groups. Adjusted means showed that managers reported higher SDE than nurses and Workers 2.

##### Impression management

The regression model for IM explained 17.7% of the variance in IM, *R*^2^ = 0.177, *F*(8,1229) = 33.11, *p* < 0.001. Higher Conscientiousness (β = 0.21, *p* < 0.001), Honesty–Humility (β = 0.20, *p* < 0.001), and Agreeableness (β = 0.06, *p* = 0.034) predicted higher IM. Neuroticism (β = −0.13, *p* < 0.001), and Extraversion (β = −0.08, *p* = 0.005) were negatively associated. Age was positively related to IM (β = 0.10, *p* < 0.001), whereas Openness and sex were non-significant.

The GLM analysis showed a smaller but significant group effect, *F*(2, 1225) = 9.98, *p* < 0.001, ηp^2^ = 0.016. Managers and nurses reported higher IM than Workers 2.

### Cross-context patterns

Across all five groups, differences in SDE were modest. Managers and inmates showed similar mean levels, whereas nurses reported somewhat lower SDE, consistent with the interpretation of SDE as a relatively stable intrapersonal self-regulatory tendency. In contrast, group differences in IM were more pronounced: managers and nurses reported higher IM, whereas inmates showed lower levels. This pattern indicates higher observed IM levels in occupational groups relative to inmates. Given the cross-sectional design, these differences may reflect contextual influences, self-selection processes, or both. Although statistically significant, group effects were small to moderate in magnitude, indicating that contextual factors may modestly shape SDR expression without altering its underlying trait structure.

### Summary of validation evidence

Across both personality frameworks, regression analyses demonstrated consistent nomological patterns supporting the construct validity of the BIDR-6 dimensions. SDE was associated with higher Extraversion and Conscientiousness and lower Neuroticism, reflecting a confident and emotionally regulated self-view. IM was positively related to Agreeableness, Conscientiousness, and was negatively related to Neuroticism (and, in the HEXACO model, Extraversion), consistent with a norm-oriented, socially regulated response style.

These associations were largely stable across occupational and correctional groups, supporting the interpretation of the BIDR-6 dimensions as trait-linked self-regulatory tendencies rather than purely context-bound response distortions.

These associations were largely stable across correctional and occupational contexts, supporting the interpretation of the BIDR-6 dimensions as trait-linked self-regulatory tendencies rather than purely context-bound response distortions.

## Discussion

### Overview and main findings

The present study examined whether the short form of the BIDR-6 reflects stable self-regulatory dispositions rather than merely response bias by evaluating its structure and construct validity across four heterogeneous groups. Across complementary analytic approaches—EGA, IRT, DIF, and CFA—the findings were broadly consistent with the theorized two-dimensional structure comprising SDE and IM. Item-level analyses indicated generally satisfactory discrimination and measurement precision, with only minor deviations (e.g., comparatively weaker parameters for BIDR8 and BIDR13) that did not compromise the overall structure.

The nomological validation phase further demonstrated theoretically coherent personality patterns. SDE was generally associated with higher Extraversion and Conscientiousness and lower Neuroticism (and, in the HEXACO framework, lower Honesty–Humility), consistent with a confident and emotionally regulated self-view. IM was positively related to Agreeableness, Conscientiousness, and Honesty–Humility, and negatively related to Neuroticism, reflecting a norm-oriented and strategically regulated self-presentation style. Importantly, these trait associations were largely consistent across correctional and occupational contexts, indicating that the BIDR-6 dimensions show stable personality linkages even in environments differing substantially in evaluative pressure.

Although modest group differences emerged—particularly for IM in occupational settings—these effects were small relative to trait-based associations and did not alter the underlying dimensional structure. Thus, contextual demands appear to shape the expression of socially desirable responding without fundamentally changing its trait-related foundations.

Extending earlier work conducted primarily in student samples, the present findings demonstrate that SDE and IM capture psychologically meaningful self-regulatory tendencies that generalize across contexts characterized by differing institutional norms and reputational stakes.

By testing both dimensional stability and nomological consistency across markedly different evaluative environments, this study directly addresses the long-standing debate over whether SDR reflects context-bound distortion or a broader self-regulatory disposition. The results support the latter interpretation while acknowledging that contextual factors may modulate the level—but not the structure—of these tendencies.

### Structure of SDE and IM as self-regulatory tendencies

Both EGA and IRT supported the distinctiveness and satisfactory functioning of SDE and IM. Their structural stability across groups spanning correctional and occupational contexts suggests that SDR cannot be reduced to a simple situational distortion. If SDR were merely context-bound faking, greater variability in dimensional structure and item functioning would be expected across environments characterized by different evaluative pressures. Instead, the two components displayed consistent organization, predictable item functioning, and limited differential item functioning.

This structural coherence aligns with theoretical perspectives that conceptualize SDE and IM as reflecting self- and social-regulatory processes rather than solely measurement artifacts (Paulhus, 2002; Uziel, 2010). SDE appears to capture relatively stable intrapersonal self-enhancement tendencies, whereas IM reflects context-sensitive interpersonal regulation aimed at maintaining social approval. The present findings therefore support the view of SDR as possessing trait-like properties while remaining responsive to contextual demands.

Most items showed adequate discrimination and limited DIF, supporting measurement robustness. SDE provided relatively uniform information across the latent continuum, whereas IM offered higher precision at lower-to-moderate levels of norm-oriented self-presentation, consistent with its regulatory function in socially evaluative contexts.

### Item-level nuance: BIDR8

Across analytic approaches, BIDR8 displayed somewhat weaker and less stable community assignment in the EGA relative to other items. However, this pattern was modest in magnitude and did not replicate consistently across bootstrap samples. Importantly, item response theory and differential item functioning analyses indicated acceptable discrimination and invariant functioning when BIDR8 was specified within the SDE dimension.

Conceptually, BIDR8 reflects comfort with social disapproval, a content domain that may tap both intrapersonal self-assurance and interpersonal considerations. Such dual relevance may render the item more sensitive to data-driven partitioning methods such as network analysis. Nonetheless, its overall psychometric performance and theoretical alignment support its placement within SDE, consistent with the original formulation by Paulhus (1991) and the short-form adaptation by Bobbio and Manganelli (2011). Taken together, these findings suggest that BIDR8 does not represent a structural anomaly but rather illustrates how certain self-assurance items may display minor method-dependent variability without undermining dimensional coherence.

### Cross-group generalizability

Patterns of structural stability and personality associations were broadly comparable across correctional and occupational groups, suggesting that SDR reflects individual-difference tendencies rather than purely situational response artifacts. This cross-context consistency directly addresses concerns raised in prior reviews regarding the robustness and generalizability of SDR measures in heterogeneous populations (e.g., Gnambs and Kaspar, 2015; Perinelli and Gremigni, 2016).

The findings align with theoretical accounts proposing that SDR represents trait-linked self-regulatory processes expressed across contexts, even if their behavioral manifestations vary depending on situational demands (Paulhus, 2002; Uziel, 2010). Evidence for substantive components in SDR has also been observed across cultural contexts (Schwartz et al., 1997), suggesting that SDR scales may capture value-linked tendencies alongside stylistic effects. Although full multi-group confirmatory invariance testing was beyond the scope of the present study, converging evidence from DIF analyses and regression models indicated minimal item bias and largely stable nomological patterns. Only one item (BIDR2) showed statistically significant DIF in discrimination, and the magnitude of this effect was small and did not alter the dimensional interpretation.

At the same time, modest group differences—particularly for IM—are consistent with research demonstrating that impression management is sensitive to evaluative pressure while retaining stable trait-like components (de Vries et al., 2013; Uziel, 2014). Thus, contextual influences appear to shape the level of expression of SDR without fundamentally altering its underlying structure.

Importantly, data were collected anonymously and without immediate evaluative consequences. The persistence of modest but systematic group differences under these conditions is consistent with the interpretation that variation in IM reflects internalized self-regulatory tendencies rather than solely reactive distortion in response to direct scrutiny.

Taken together, these results support a balanced interpretation: SDR reflects enduring self- and social-regulatory tendencies that generalize across diverse institutional contexts while remaining responsive to normative and evaluative demands.

### Personality correlates and functional interpretation

As anticipated, SDE was generally associated with lower Neuroticism and higher Extraversion and Conscientiousness, replicating patterns reported in earlier work (Paulhus, 2002; Paulhus and John, 1998). The Big Five regression results align with prior research showing that SDE reflects a self-confident, achievement-oriented self-view characterized by higher Extraversion and Conscientiousness, coupled with lower Agreeableness (e.g., Bäckström et al., 2009; Paulhus, 2002). The absence of substantial of group differences suggests that these personality correlates of SDE generalize across occupational and correctional groups. Together, these associations indicate that SDE reflects a confident and emotionally regulated self-view rather than deliberate deception.

IM positively related to Agreeableness, Conscientiousness, and Honesty–Humility, consistent with prior findings linking IM to norm adherence and socially regulated behavior (de Vries et al., 2013; Uziel, 2014). The pattern of Big Five regression results further supports the interpretation of IM as reflecting a socially compliant and self-controlled orientation characterized by higher Agreeableness and Conscientiousness, and lower Neuroticism (Bäckström et al., 2009; Paulhus, 2002; Zettler et al., 2015), with these associations largely comparable across occupational and correctional groups. Importantly, such associations do not imply that IM represents honest responding. Rather, as Paulhus (1984, 2002) emphasized, IM reflects a tendency toward socially regulated self-presentation. The present findings do not permit direct inference regarding strategic intent, but they are consistent with IM reflecting norm-oriented response tendencies associated with social adaptation.

The somewhat stronger SDE–Neuroticism association among inmates suggests that self-enhancing biases may serve compensatory regulatory functions under high stress or institutional constraint.

Taken together, these findings support conceptualizing SDR as a set of self- and social-regulatory dispositions rather than merely a threat to validity.

Prior studies have examined the nomological network of the BIDR. The present contribution extends this work by (a) integrating modern psychometric approaches (EGA, IRT, DIF) with construct validation, (b) testing cross-context robustness across correctional and occupational settings, and (c) examining item-level functioning within these diverse environments.

### Limitations and future directions

Several limitations warrant consideration. First, the cross-sectional design precludes causal inference and does not allow direct examination of temporal stability or regulatory dynamics. Longitudinal research would be valuable for clarifying how SDE and IM unfold over time and in response to changing evaluative contexts.

Second, all constructs were assessed via self-report measures. Although this is standard in SDR research, reliance on a single method may inflate shared variance. Future studies would benefit from multimethod approaches, including informant reports, behavioral indicators, or experimental manipulations of evaluative pressure.

Third, while pooling of groups was supported by minimal differential item functioning and broadly comparable structural patterns, full multi-group measurement invariance testing was not conducted. More stringent invariance analyses across correctional and occupational contexts would provide additional evidence regarding cross-context comparability.

Fourth, although the BIDR-6 demonstrated generally satisfactory psychometric performance, overall model fit indices were modest, and minor item-level nuances (e.g., comparatively weaker parameters for BIDR8) were observed. Future research may further refine short-form versions through alternative wording, cross-validation, or item-level optimization.

Fifth, the occupational groups were predominantly female, whereas the inmate group was predominantly male. Although sex was statistically controlled in regression and GLM analyses, this imbalance may limit generalizability and warrants consideration in future research.

Finally, the study was conducted within a single national context. Replication across cultural settings is necessary to determine whether the observed structure and nomological patterns generalize across differing normative and institutional environments.

## Conclusion

This study provides convergent evidence that the BIDR-6 is a structurally coherent and theoretically interpretable measure of socially desirable responding. A two-factor structure— Self-Deceptive Enhancement and Impression Management—was supported across complementary psychometric approaches and diverse populations. Both dimensions demonstrated meaningful and theoretically consistent associations with major personality traits, reinforcing their interpretation as trait-linked self-regulatory processes.

Although minor item-level nuances and modest contextual differences were observed, the overall pattern indicates that socially desirable responding reflects relatively stable self- and social-regulatory tendencies that generalize across correctional and occupational settings rather than merely context-bound response distortion.

## Data Availability

The dataset supporting the conclusions of this article is publicly available in Mendeley Data. The repository contains the data required to replicate the psychometric analyses (EGA, IRT, CFA, and DIF) reported in this study. The dataset can be accessed at: Daderman, A. M., & Pennbrant, S. (2025). BIDR6 (Version 2) [Data set]. Mendeley Data. https://doi.org/10.17632/5h9tmc5rn2.2.
