# Supplementary material for: Revisiting the balanced inventory of desirable responding: psychometric structure and personality correlates across heterogeneous groups
Source: Front Psychol. 2026 Apr 24;17:1788770. doi: 10.3389/fpsyg.2026.1788770 (PMC13154599; doi:10.3389/fpsyg.2026.1788770)
Supplement: Supplementary file 1 [file Table_1.docx]

**Supplementary Table S1**

**Revisiting the Balanced Inventory of Desirable Responding: Psychometric Structure and Personality Correlates Across Heterogeneous Groups**

This supplementary material provides supplementary Table S1. It contains an additional information corresponding to Table 2 of the manuscript.

**Table S1.** Item-level parameters and model-fit statistics for the BIDR-6 estimated using the graded response model.

| **Dimension / Item** | **Maxvi** | **#zsig** | **S-χ² (df)** | ***p*** | ***a* / SE** | **Interpretation** | ***b*_1_** | ***b*_2_** | ***b*_3_** | ***b*_4_** | ***b*_5_** | ***b*_6_** | **λ** |
| --- | --- | --- | --- | --- | --- | --- | --- | --- | --- | --- | --- | --- | --- |
| **SDE** |  |  |  |  |  |  |  |  |  |  |  |  |  |
| BIDR7 | 0.05 | 0 | 117.25 (134) | .848 | 1.91 / 0.10 | Very high | −2.72 | −1.73 | −0.86 | 0.08 | 1.21 | 2.45 | .75 |
| BIDR2 | 0.05 | 0 | 148.31 (147) | .455 | 1.45 / 0.07 | High | −3.15 | −1.99 | −1.02 | 0.00 | 1.05 | 2.72 | .65 |
| BIDR6 | 0.04 | 0 | 130.06 (150) | .879 | 1.42 / 0.07 | High | −2.84 | −1.84 | −0.94 | 0.40 | 1.42 | 2.70 | .64 |
| BIDR4 | 0.07 | 0 | 195.74 (149) | .006 | 1.28 / 0.07 | Moderate | −2.05 | −0.82 | −0.05 | 0.96 | 2.02 | 3.50 | .60 |
| BIDR5 | 0.05 | 2 | 201.08 (153) | .006 | 1.19 / 0.06 | Moderate | −2.24 | −0.79 | 0.16 | 1.14 | 2.10 | 3.73 | .57 |
| BIDR1 | 0.05 | 0 | 181.39 (149) | .036 | 0.98 / 0.06 | Moderate | −5.21 | −3.62 | −2.31 | −0.63 | 0.97 | 3.21 | .50 |
| BIDR3 | 0.07 | 2 | 165.96 (161) | .378 | 0.97 / 0.06 | Moderate | −3.10 | −1.25 | −0.19 | 0.89 | 2.00 | 3.52 | .50 |
| BIDR8 | 0.10 | 3 | 202.13 (173) | .064 | 0.67 / 0.05 | Moderate | −5.52 | −2.91 | −1.71 | −0.57 | 0.82 | 2.56 | .36 |
| **IM** |  |  |  |  |  |  |  |  |  |  |  |  |  |
| BIDR10 | 0.04 | 0 | 252.16 (164) | < .001 | 2.05 / 0.11 | Very high | −2.55 | −1.90 | −1.28 | −0.78 | −0.36 | 0.75 | .77 |
| BIDR9 | 0.00 | 0 | 193.76 (168) | .085 | 1.96 / 0.10 | Very high | −2.04 | −1.43 | −0.75 | −0.17 | 0.28 | 1.38 | .76 |
| BIDR15 | 0.00 | 0 | 179.83 (172) | .325 | 1.28 / 0.08 | Moderate | −1.70 | −1.28 | −0.99 | −0.75 | −0.61 | 0.04 | .60 |
| BIDR16 | 0.03 | 0 | 169.89 (186) | .796 | 1.13 / 0.07 | Moderate | −3.46 | −2.67 | −1.93 | −1.13 | −0.75 | 0.34 | .55 |
| BIDR14 | 0.04 | 0 | 220.10 (181) | .025 | 1.03 / 0.06 | Moderate | −1.40 | −0.22 | 0.46 | 1.15 | 1.59 | 2.82 | .52 |
| BIDR12 | 0.06 | 0 | 264.91 (203) | .684 | 0.94 / 0.06 | Moderate | −3.25 | −2.12 | −1.22 | −0.33 | 0.30 | 2.04 | .48 |
| BIDR11 | 0.00 | 0 | 238.04 (200) | .034 | 0.79 / 0.05 | Moderate | −3.05 | −1.82 | −1.06 | −0.33 | 0.32 | 2.42 | .42 |
| BIDR13 | 0.11 | 8 | 382.00 (210) | < .001 | 0.56 / 0.05 | Low | -5.30 | -2.65 | -1.13 | 0.41 | 1.75 | 4.78 | .31 |

**Note.** *N* = 1,887. BIDR-6 = Balanced Inventory of Desirable Responding; SDE = Self-Deceptive Enhancement; IM = Impression Management.

Parameters were estimated using the graded response model in IRTPRO 4.2. λ = standardized factor loading. Maxvi and #zsig indices assessed monotonicity (Mokken package, R). Items with mild local dependence or lower discrimination were retained based on conceptual relevance and nonredundant wording. Interpretive categories for the discrimination parameter (*a*) follow the same conventions as in Table 2 of the main manuscript.

**Model-fit indices:**
SDE—AIC = 50,413.82; BIC = 50,724.21; M₂(1000) = 3,015.92, *p* < .001; RMSEA = .03.
IM—AIC = 50,786.72; BIC = 51,097.11; M₂(1000) = 3,298.75, *p* < .001; RMSEA = .03.

AIC = Akaike information criterion; BIC = Bayesian information criterion; *M*_2_ = limited-information goodness-of-fit statistic.
