# Supplementary material for: Revisiting the balanced inventory of desirable responding: psychometric structure and personality correlates across heterogeneous groups
Source: Front Psychol. 2026 Apr 24;17:1788770. doi: 10.3389/fpsyg.2026.1788770 (PMC13154599; doi:10.3389/fpsyg.2026.1788770)
Supplement: Supplementary file 2 [file Data_Sheet_1.pdf]

Supplementary Material S1

Revisiting the Balanced Inventory of Desirable Responding: Psychometric Structure and Personality Correlates Across Heterogeneous Groups

Authors:
Journal: ***Personality and Individual Differences***

This Supplementary Material provides additional information on the four samples included in the study: inmates, managers, nurses, and working adults. The descriptions clarify recruitment procedures, data collection, and sample characteristics corresponding to Table 1 of the manuscript.

Inmates

Sample

The inmate sample consisted of 287 incarcerated persons from multiple prisons in southern Sweden, representing all three Swedish security classifications (1 = highest security; 2 = medium; 3 = lowest). Most participants were housed in security class 2 facilities. Sentences ranged from short-term incarceration to life imprisonment. Only inmates fluent in Swedish were invited to participate.

Procedure

Data were collected in 2016–2017. Data collection among inmates was coordinated by the research team. After approvals were obtained from university authorities and the Swedish Prison and Probation Service, questionnaire packages were mailed to participating prisons. Due to security restrictions, prison staff photocopied questionnaires and coordinated internal distribution. In several facilities, inmate representatives helped distribute and collect surveys; in others, correctional officers handled distribution.

Participation was voluntary and anonymous. Inmates received written information about the study's research purpose, confidentiality, and ethics. Completed questionnaires were sealed in envelopes and returned by mail. Small phone-card incentives were provided for participation and assistance with distribution. Of 300 distributed questionnaires, 287 were returned (response rate = 95.7%).

Workers 1 (Control Group for Inmates)

Sample

The control sample consisted of 162 working adults enrolled in a university course. Most were professionals in fields such as psychology, social work, policing, law, medicine, or corrections.

Procedure

Data were collected in 2018. Data for workers 1 were collected as part of coursework research activities at the home institution. At course introduction, students were invited to participate and provide email addresses for contact. Those who agreed received information about the project and were told that their data would function as a comparison group for the inmate

study. They completed the same questionnaires used in the inmate sample. Of 422 invitations, 162 participants responded (response rate = 38.4%).

Managers

Sample

The manager sample included 344 managers from nine organizations in Sweden. Approximately 70% worked in human-service sectors (e.g., social services, healthcare, education) and 30% in manufacturing or industrial settings. Participants represented private companies (45%) and municipal/state organizations (55%). Managerial levels ranged from senior (19.4%) to intermediate (68.6%) and first-line leaders (12%).

Procedure

Data were collected as part of a leadership project conducted in 2017. HR departments in participating organizations distributed study information to CEOs, who approved participation. HR then provided mailing lists or internal distribution mechanisms. Invitations containing study descriptions, ethical information, and a link to the anonymous online survey (Google Forms) were sent via email.

Three reminder emails were issued to maximize participation. Response rates within organizations ranged from 65–81%, averaging 73%. Data collection lasted approximately five weeks.

Nurses

Sample

The nurse sample consisted of 939 registered nurses (RNs) employed in hospital and primary healthcare settings in western Sweden. The gender distribution (88.5% women) mirrored national nursing statistics. Nearly half were specialist nurses, indicating a well-educated and experienced cohort.

Procedure

Data were collected in 2016–2018. Data collection occurred in two stages. First, questionnaires were distributed to RNs enrolled in continuing-education courses at a Swedish university (~120 responses). To obtain a larger sample, the research team later collaborated with regional healthcare authorities, who provided email lists of employed RNs.

Nurses received an email invitation with study information, ethical details, and a link to the anonymous online questionnaire (Google Forms). Three reminders were issued due to the anonymous design. In total, 1,450 nurses were invited, and 939 responded (response rate = 64.8%).

Workers 2 (General Working Adults)

Sample

The workers 2 sample comprised 171 adults with work experience (two responses excluded for missing data). Most participants were employed or self-employed (80%), held a university degree (57%), and were in a relationship (75%). Work experience ranged from 1–64 years (M = 20.11). A subset (n = 29) held managerial roles.

Procedure

Data were collected in 2019 via an online survey (Google Forms). Data for workers 2 were obtained via an independent student project conducted under university supervision. Recruitment relied on convenience and snowball sampling through the researchers' social media networks, contacts in municipalities, and links shared within private-sector organizations. QR codes were later distributed to broaden reach.

Participants received an information letter describing the study, voluntary participation, anonymity, and ethical principles. No incentives were offered. Because of anonymous distribution, a formal response rate could not be calculated. After excluding two incomplete cases, 171 responses were analyzed.

Summary of Procedures Across Samples

Across all samples, participation was voluntary, anonymous, and based on written informed consent. With the exception of the inmate sample—which required paper-and-pencil administration due to institutional security procedures—all data were collected through self-administered online surveys (Google Forms). Participants in the manager, nurse, and working-adult samples received identical ethical information, including descriptions of study purpose, confidentiality safeguards, and anonymity. In these three groups, data collection followed a consistent procedure involving email invitations, detailed study information, and three reminder messages to maximize participation. Despite differences in recruitment sources and organizational settings, the overall methodological approach was comparable across non-incarcerated samples, ensuring uniform administration and ethical standards.
